# Juvenile systemic lupus erythematous in Iranian children

**DOI:** 10.1186/1546-0096-12-S1-P330

**Published:** 2014-09-17

**Authors:** Fatemeh Tahghighi, Vahid Ziaee, Mohamad-Hassan Moradinejad, Fatemeh Fereshteh Mehregan

**Affiliations:** 1 Division of pediatric Rheumatology, Children’s Medical Center, Tehran University of Medical Sciences, Tehran, Iran, Islamic Republic Of

## Objectives

Juvenile systemic lupus erythematous (JSLE) is a multi-system autoimmune disorder of unknown origin. Involvement of the mucocutaneous ,musculoskeletal system and kidney disease are the most common manifestation of JSLE. This investigation was performed to define the demography and clinical manifestation of JSLE in Iran.

## Methods

Fifty nine patients with JSLE were enrolled in this retrospective study. All patients fulfilled the American College of Rheumatology revised criteria 1982 for the diagnosis of SLE and had shown clinical manifestations of the disease before the age of 16.

## Results

A total of 59 patients, 49 (89%) were female and10 (16.9%) male. The female to male ratio was 4.9:1. The mean age of onset was 10.5 years (range 2-16). The mean duration of disease from diagnosis was 2 years (range7 months-5 years). The most common of constitutional sign was fatigue (76.3%) and the other signs were fever (32.2%), Nocturnal pain (20.3%), Mood disorder (20.03%), and Weight loss (22%). The most common manifestation of JSLE were as follows: 48 patients (81.35%) had mucocutaneous involvement, 46 patients (77.9%) had musculoskeletal involvement, 25 children (42.37%) suffered from renal disease, hematological abnormalities were detected in 19 patients (32.2%), 10 patients (16.94%) had cardiovascular disease, 33 patients (55.93%) presented nervous system involvement, 12 patients (20.33%) had pulmonary disease, and 13 patients (22.03) experienced infection complications. During the follow up period(3years) two patients died, one from renal failure, one from infections.

## Conclusion

A high index of suspicion must be preserved for the diagnosis of SLE in adolescent children, particularly girls. Juvenile systemic lupus erythematous (JSLE) is a critical multi-system organ disease. JSLE still has a significant mortality rate high, although it has a high remission rate with early diagnosis and treatment.

## Disclosure of interest

None declared

